# The State Doctor and the Home

**Published:** 1921-01-29

**Authors:** 


### The State Doctor and the Home.

*To the Editor of* The Hospital.

Sir,—The Hospital does wisely to ask the opinions of those who are mainly to be affected by the new organisation of the Ministry of Health. It would be excellent if views could be obtained from such bodies as women's institutes, mothers' unions, and co-operatives, and from the men's discussions at Chapel Brotherhood and Church of England Men's Society meetings, for there the working man and woman have been learning to utter a considered opinion with courage, candour, and courtesy. But, as your leader says, it is difficult to see this wood for its trees—the discussion of subsidiary points has sadly obscured the issue. As regards the working woman, she has now been told over and over again about the need of good citizens and the seriousness of our present death-rate and damage-rate. She is rather overwhelmed, however, by the multitude of counsellors and authorities; even if her child breaks the law the latest theory requires that he shall be medically examined afresh to see whether delinquency is due to physical disease. But she would very much like to bring up all her children—let them only not be too many to bring up—for, despite all that is said of her indifference, little white coffins do wound a mother's heart. If they do not, it may be that repeated blows and the grinding of poverty have made it callous.

If the working mother speaks her mind, she will voice some distrust of a record kept on a card or a *dossier* jotted by successive practitioners, who have not watched as a "family doctor" watches the patient as one of a family whose idiosyncrasies he knows, has known, perhaps, through two generations. From his pre-natal days at the mother-school our young citizen will pass through the hands of—how many doctors? While, incidentally, a small army of officials, school nurse, health visitor, Care Committee worker, will throw in counsels for his welfare. The friendly chemist, who so often gave a poor woman "summat" for her child's cough or rheumatism each November, really knew more of the family idiosyncrasies and tendencies than these birds of passage. The ideal State service, which would be preventive, is really a scheme by which each household would be visited by its doctor-friend, as a good clergyman visits his parishioners. Special troubles he would refer to a specialist, early symptoms he would note, wise incidental counsels about ventilation and sanitation he—or very usefully she—would give without incurring resentment. The home is really a far better place than the school for the examination of the school-child, and if the indifference or carelessness of a mother prevented the carrying out of medical orders, there should be the district nurse to fall back upon.

As to the free choice of doctor, one consideration is important. It is that if uneducated people do not like and trust their medical adviser he will not get the truth from them. The consideration that his diagnosis must be damaging and may be futile if they keep back information does not weigh with them as with educated people. The mission of the doctor must in the end become the Chinese one—to keep people well rather than to cure them when ill. That is a point which appeals to the wage-earner.—

I am, Sir, yours faithfully, A Social Worker.

